# Prevalence, high-risk HPV type profile, and factors associated with high-risk HPV infection among women living with HIV in Kinshasa, Democratic Republic of the Congo

**DOI:** 10.3389/fpubh.2026.1914799

**Published:** 2026-09-18

**Authors:** Julien Neze-Sebakunzi, Anna Maria Doro Altan, Susanna Ceffa, Giovanni Guidotti, Stefano Capparucci, Fausto Ciccacci, Marlène Musikingala, Antoine Nkuba-Ndaye, Jean-Claude Makangara-Cigolo, Jean-Paul Kompany Kisenzele, Trésor Kabeya-Mampuela, Stefano Orlando, Steve Ahuka-Mundeke

**Affiliations:** 1 Service of Microbiology, Department of Medical Biology, Kinshasa University Hospital, University of Kinshasa, Kinshasa, Democratic Republic of Congo; 2 Community of Sant'Egidio, DREAM Program DRC, Kinshasa, Democratic Republic of Congo; 3 Università degli Studi Link, Rome, Italy; 4 Community of Sant'Egidio, DREAM Program Europe, Rome, Italy; 5 Azienda Sanitaria Locale (ASL) Roma 1, Rome, Italy; 6 Department of Biomedicine and Prevention, University of Rome Tor Vergata, Rome, Italy

**Keywords:** cervical cancer, Democratic Republic of the Congo, high-risk HPV, HIV, human papillomavirus (HPV), Kinshasa, women living with HIV

## Abstract

**Background:**

Cervical cancer is one of the most common cancers in women, particularly among women living with HIV. High-risk human papillomavirus (Hr-HPV) is the main causative agent of this cancer, but the prevalence of Hr-HPV infection among women living with HIV in Kinshasa remains poorly documented. The objective of this study was to determine this prevalence and identify associated risk factors.

**Methods:**

A cross-sectional study was conducted among women living with HIV aged 25–65 years receiving antiretroviral therapy at the DREAM Center in Kinshasa. Participants were consecutively recruited among eligible women attending the center during the study period. Cervical samples were collected using the Abbott Cervi–Collect kit and analyzed for Hr-HPV genotyping using the Abbott RealTime HPV assay on the m2000 platform, a real-time PCR method detecting HPV-16, HPV-18, and a pooled group of 12 additional high-risk HPV types with an internal β-globin control for sample quality assurance. Sociodemographic and clinical data were collected using structured questionnaires.

**Results:**

A total of 436 women were included in the analysis. The prevalence of Hr-HPV infection was 47.7%. Of these, 23.79 % were infected with oncogenic HPV-16 and−18 types (alone or in co-infection with other types). In a logistic regression analysis, WHO clinical stage 3 and 4, duration of antiretroviral therapy for less than 2 years, and HIV viral load greater than or equal to 1,000 copies/ml showed statistically significant associations with Hr-HPV infection, with adjusted ORs of 1.75 (1.16–2.64; *p* = 0.008), 1.91 (1.07–3.41; *p* = 0.028), and 3.13 (1.30–7.54; *p* = 0.011), respectively.

**Conclusions:**

This study reveals a high prevalence of Hr-HPV infection among women living with HIV in Kinshasa. Non-16/18 HPV types were predominant, while HPV-16 and HPV-18 were detected in approximately one in four Hr-HPV-positive women. Advanced HIV disease (WHO clinical stages 3 and 4), unsuppressed HIV viral load, and shorter duration of antiretroviral therapy were independently associated with Hr-HPV infection. These findings underscore the potential value of broader HPV vaccine coverage, including higher-valent vaccines, alongside strengthened cervical cancer screening as part of comprehensive cervical cancer prevention strategies.

## 1 Introduction

Cervical cancer (CC), classified as an AIDS-defining cancer (1, 2), constitutes a major public health problem (3, 4). It is one of the leading causes of cancer mortality worldwide and ranks as the fourth most common cancer in women (1, 5–7). In 2020, approximately 604,000 women were diagnosed with CC, resulting in approximately 342,000 deaths (6). Nearly 90% of CCs occur in low- and middle-income countries (1, 3, 7, 8), where it is often the leading or second leading cause of cancer and death in women (7, 9). In the Democratic Republic of the Congo (DRC), the incidence of CC is high, surpassing that of breast cancer (10, 11).

The main causative agent of CC is the human papillomavirus (HPV) (3, 6, 7, 12). Genital HPV infection is the most common sexually transmitted infection (STI) worldwide (13, 14), with some researchers calling it the “sex flu” (13). Sexually active women are particularly at risk, and the persistence of this infection is critical for the development of CC (1, 12, 15). Approximately 200 HPV species have been identified (16), and are classified into low-risk (Lr-HPV) or high-risk (Hr-HPV) subtypes based on their oncogenic potential (8, 11). Low-risk types can be asymptomatic or cause anogenital warts, while high-risk types are the cause of CC (3). Types 16 and 18 are responsible for approximately 70% of CC cases worldwide (1, 17, 18). Women living with HIV (WLHIV) are at increased risk of precancerous lesions and cervical cancer compared to HIV-negative women (2, 4, 13, 19). HIV-induced immunosuppression is associated with a higher prevalence of HPV, including high-risk types (9, 20). HPV infections in HIV-positive women are not only more frequent, but they also persist more often, leading to an increased prevalence of high-grade cervical lesions (2, 9). Unlike some opportunistic infections, such as Pneumocystis jiroveci pneumonia, extrapulmonary cryptococcosis, or histoplasmosis, whose incidence has decreased after the introduction of antiretroviral therapy, the incidence of CC has not decreased significantly. This is because persistent immunosuppression, a characteristic feature of people living with HIV, remains a major risk factor for the development of virally related cancers, including HPV (3). The risk of developing cervical cancer is six times higher in WLHIV than in those who are not infected (18, 21).

In high-income countries, cervical cancer incidence and mortality have fallen by more than 50% over the past three decades, thanks to the implementation of national screening programs. On the other hand, in low- and middle-income countries, the absence or weakness of screening and vaccination initiatives against human papillomavirus (HPV) continues to contribute to the persistence of this public health problem (3).

CC is largely preventable through vaccination and screening for precursor lesions, with appropriate follow-up and treatment (4). Early screening of women at risk of developing cervical lesions is essential to reduce the incidence of CC (6). As part of the second edition of the World Health Organization (WHO) guidelines, published in July 2021 for the screening and treatment of precancerous cervical lesions, it is now recommended to prioritize HPV deoxyribonucleic acid (DNA) detection as the primary screening test, replacing visual inspection with acetic acid (VIA) or cytology, both for the general population and for women living with HIV (18). Indeed, recent research has explored the use of HPV testing as a primary screening modality, demonstrating that it is as effective, or even more than cytology alone (8, 18).

However, although a 2019 study reported a prevalence of 24.4% of Hr-HPV infections in the general female population of Kinshasa (3), there is a lack of specific data regarding WLHIV in this region.

Therefore, our study aims to estimate the prevalence and distribution of Hr-HPV infections among women living with HIV in Kinshasa, while identifying the factors associated with these infections.

## 2 Methods

### 2.1 Study type, setting, and population

We conducted a single-center observational cross-sectional study from April 2022 to April 2023. The study population included women aged 25 to 65 years receiving antiretroviral therapy (ART) at the DREAM Center in Kinshasa, located in the municipality of N'Sele, Kinshasa. The DREAM Center is a specialized facility providing HIV care and treatment free of charge and serves patients from N'Sele as well as from other municipalities of Kinshasa. Women were invited to participate and were enrolled progressively, based on their attendance at scheduled medical check-up appointments during the study period. To limit non-inclusion bias, all women who missed their appointments received phone call reminders to encourage them to attend their appointments and participate in the screening.

Informed consent was obtained from all participants. Pregnant women were excluded according to the predefined eligibility criteria of the study. Women with a history of hysterectomy, those who had previously undergone HPV testing, and those who had already received HPV vaccination were also excluded. For women who were menstruating at the time of the visit, sample collection was deferred until the end of menstruation to ensure standardized sampling conditions.

The required sample size (*n*) is at least 385 participants. It was calculated assuming a prevalence of 50% due to the absence of precise data, a 95% confidence interval (with a Z-score of 1.96), and a precision of 5%:


$$\begin{array}{l} \text{n}=\left({\text{Z}}_{-}{\left(1-\alpha /2\right)}^{2}\text{ X P }\left(1-\text{P}\right)\right)/\text{i} \end{array}$$

### 2.2 Data collection and laboratory procedures

Data were collected using a structured and standardized questionnaire developed specifically for this study and pretested before data collection. The questionnaire was interviewer-administered and covered demographic variables (age, provenance, education, employment status, marital status), reproductive factors (contraceptive use, age at first intercourse, parity, number of lifetime sexual partners), and clinical characteristics (weight and height for body mass index (BMI)).

For the purposes of this study, the variable “provenance” was categorized into two groups: “intrazonal” and “extrazonal.” “Intrazonal” refers to participants residing within the municipality where the health facility is located (the municipality of N'Sele), whereas “extrazonal” refers to participants residing outside this municipality. Provenance was included as an exploratory variable to assess whether geographical origin was associated with Hr-HPV infection. It is important to note that the municipality of N'Sele is considered an urban–rural area of Kinshasa. Additionally, WHO clinical stage and ART duration were extracted from patients' electronic medical records.

For HIV viral load (VL), venous blood was collected in Ethylene Diamine Tetraacetic Acid (EDTA) tubes, plasma separated by centrifugation, and stored at −20 °C. VL was measured using Abbott RealTime HIV-1 on the m2000 platform (Abbott Molecular, Abbott Park, IL, USA), following manufacturer's protocols. RNA was extracted from 0, 6 ml of plasma using magnetic particle technology and amplified by PCR (22).

A cervical sample was collected using the Abbott Cervi-Collect^®^ Specimen Collection Kit (Abbott Molecular, Wiesbaden, Germany), following the manufacturer's instructions. After insertion of the cervical brush into the cervical os, it was gently rotated three times in a single direction along the endocervical mucosa, and the collected material was then transferred into a transport medium tube.

For women who were menstruating at the time of the visit, sample collection was deferred until the end of menstruation to ensure standardized sampling conditions.

Samples were then stored at 2 °C−8 °C for up to 2 weeks at the DREAM Laboratory in Kinshasa to standardize storage conditions across all samples, in accordance with the manufacturer's instructions, which allow storage between 2 °C and 30 °C for up to 14 days.

HPV genotyping was performed using the Abbott RealTime HPV assay on the m2000 platform (Abbott Molecular, Abbott Park, IL, USA). The assay is based on a multiplex real-time PCR targeting the L1 region (GP5+/6+) of the viral genome. Following automated DNA extraction according to the manufacturer's instructions, amplification and detection were performed simultaneously using fluorescent probes. The assay detects HPV16 and HPV18 individually, and pools 12 other high-risk HPV genotypes (HPV31, −33, −35, −39, −45, −51, −52, −56, −58, −59, −66, and −68). An internal β-globin control and external positive and negative controls ensured sample adequacy and assay validity (23).

### 2.3 Statistical analysis

Data processing and analysis were performed using Stata^®^ 14 (StataCorp, 2014). The prevalence of Hr-HPV was estimated with 95% confidence intervals (CI). Categorical variables were presented as proportions and continuous variables were summarized as mean ± standard deviation (SD) for normally distributed data, or as median and interquartile range (IQR) for skewed distributions. The normality of the data was assessed both visually, using density curves overlaid on histograms, and statistically, using the Shapiro–Wilk test. Univariate and multivariate logistic regression models were used to identify factors associated with Hr-HPV among WLHIV. Variables with *p* < 0.2 in univariate analysis or known relevance based on existing literature were included in the multivariate logistic regression model. Adjusted odds ratios (aOR) with 95% CI were calculated to assess associations with Hr-HPV infection; *p* < 0.05 was considered significant. Multicollinearity among independent variables was assessed using variance inflation factors (VIF). A VIF value >5 was considered indicative of problematic multicollinearity. Model fit was assessed using the Hosmer–Lemeshow goodness-of-fit test.

### 2.4 Ethical considerations

This study was conducted in accordance with the Declaration of Helsinki and approved by the Ethics Committee of the School of Public Health, University of Kinshasa (ESP/CE/31/2022, April 27, 2022). All participants provided written informed consent and were free to withdraw at any time without any consequences. Data collected were treated with strict confidentiality and anonymity. Participants received their test results free of charge, and those who tested positive were referred for further clinical evaluation and appropriate management.

## 3 Results

### 3.1 Study participant flow

Of the 488 HIV-positive women aged 25–65 years and receiving antiretroviral therapy, 451 were enrolled in the study. The remaining 37 were not enrolled for the following reasons: 23 women were pregnant, 1 woman had a history of hysterectomy, and 13 refused consent. For participants who were menstruating at the time of the study visit, sample collection was postponed until the end of the menstrual period. Among the 451 enrolled women, 15 were excluded from the analysis: 11 for refusal to sample (due to fear or lack of preparation) and 4 others for bleeding observed during the attempted cervical sampling. Consequently, a total of 436 women were included in the study (Figure 1).

**Figure 1:**
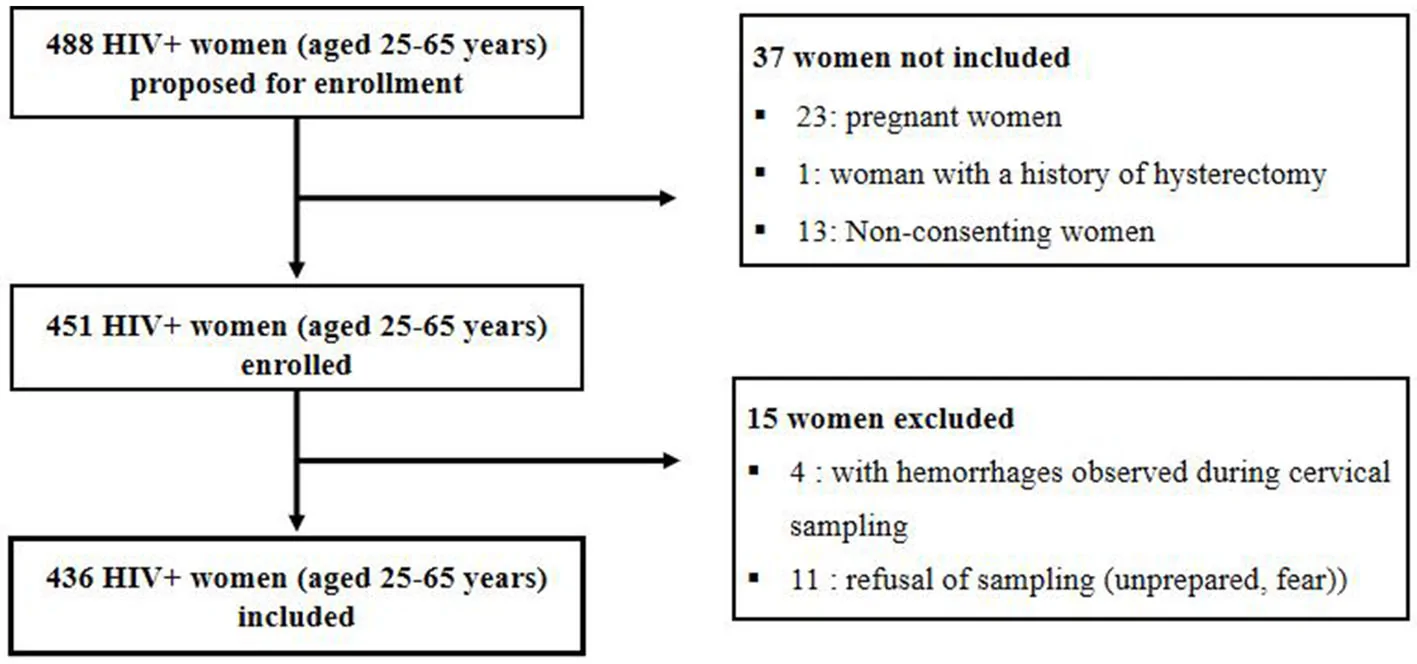
Participant flow diagram.

### 3.2 Demographic, reproductive, clinical, and biological characteristics

The mean age of participants was 44.46 years (± 9.02). Women aged 25–29 had the highest positivity rate for Hr-HPV (60%), while older age groups showed a decrease, reaching 43.79% among those aged 40–49. Women living outside the N'sele health zone had a slightly lower positivity rate (45.54%) than those living in this zone (49.06%). The rate was lowest among those with a university degree (37.50%). Skilled workers and women in a relationship had lower positivity rates (43.75 and 40.11%) than those unemployed or single (Table 1).

**Table 1:** Sociodemographic characteristics and Hr-HPV screening results among WLHIV, aged 25–65, receiving antiretroviral therapy at the DREAM Center in Kinshasa, Democratic Republic of Congo [*n* = 436].

Characteristics	Hr-HPV		
	Total (*N*)	Positive [*n* (%)]	Negative [*n* (%)]
Age range (years) *[Mean (SD): 44.46 (±9,02)]*			
25 – 29	25	15 (60.00)	10 (40.00)
30–39	111	55 (49.55)	56 (50.45)
40–49	169	74 (43.79)	95 (56.21)
50–59	109	50 (45.87)	59 (54.13)
60–65	22	12 (54.55)	10 (45.45)
Provenance^*^			
Extrazonal	224	102 (45.54)	122 (54.46)
Intrazonal (N'sele health zone)	212	104 (49.06)	108 (50.94)
Level of education			
None or elementary	98	47 (47.96)	51 (52.04)
High school	298	144 (48.32)	154 (51.68)
University	40	15 (37.50)	25 (62.50)
Employment status			
None/unemployed	209	100 (47.85)	109 (52.15)
Unskilled worker	179	85 (47.49)	94 (52.510)
Skilled worker	48	21 (43.75)	27 (56.25)
Marital status			
Single (single - divorced - widow)	259	135 (52.12)	124 (47.88)
In a relationship (married - cohabitation)	177	71 (40.11)	106 (59.89)

Hr-HPV, high risk human papillomavirus, SD, standard deviation, %, percentage.

^*^Provenance: intrazonal refers to participants residing within the pality where the health facility is located (municipality of N'Sele, an urban–rural area). Extrazonal refers to participants coming from outside this municipality.

Among WLHIV included in the study, the proportion of Hr-HPV infection was higher among those with advanced HIV disease (WHO clinical stages 3 and 4) than among those in stages 1 and 2 (55.1 vs. 38.8%). The prevalence also increased with HIV viral load, from 44.4% among women with a viral load <40 copies/ml to 71.4% among those with a viral load ≥1,000 copies/ml. Similarly, women receiving antiretroviral therapy for less than two years had a higher prevalence of HR-HPV infection than those treated for at least two years (61.5 vs. 44.7%). In contrast, the prevalence of Hr-HPV infection showed little variation according to contraceptive use, age at **first** sexual intercourse, number of sexual partners, or parity (Table 2).

**Table 2:** Clinical, reproductive, and laboratory characteristics and Hr-HPV screening results among WLHIV, aged 25–65 years, receiving antiretroviral therapy at the DREAM Center in Kinshasa, DRC [*n* = 436].

Characteristics	Hr-HPV		
	Total (N)	Positive [*n* (%)]	Negative [*n* (%)]
Contraceptive use			
None	384	181 (47.14)	203 (52.86)
Hormonal	52	25 (51.92)	27 (48.08)
Age at first sexual intercourse (years) *[Median (IQR): 17 (15–18)]*			
≤ 15	142	69 (48.59)	73 (51.41)
16–18	229	112 (48.91)	117 (51.09)
>18	65	25 (38.46)	40 (61.54)
Number of sexual partners (lifetime)			
<4	185	84 (45.41)	101 (54.59)
4–7	154	70 (45.45)	84 (54.55)
≥8	97	52 (53.61)	45 (46.39)
Parity *[Median (IQR): 4 (2–6)]*			
≤ 4	258	116 (44.96)	142 (55.04)
>4	178	90 (50.56)	88 (49.44)
BMI (Kg/m^2^) *[Median (IQR): 23.53 (20.55–27.60)]*			
<18.5	49	31 (63.27)	18 (36.73)
18.5– <25	218	104 (47.71)	114 (52.29)
≥25– <30	112	48 (42.86)	64 (57.14)
>30	57	23 (40.35)	34 (59.65)
WHO Clinical Stage (HIV)			
1 & 2	209	81 (38.76)	128 (61.24)
3 & 4	227	125 (55.07)	102 (44.93)
HIV Viral load (copies/ml)			
<40	358	159 (44.41)	199 (55.59)
40– <1,000	50	27 (54.00)	23 (46.00)
≥1,000	28	20 (71.43)	8 (28.57)
Duration of ART (years)			
<2 ans	65	40 (61.54)	25 (38.46)
≥2 ans	371	166 (44.74)	205 (55.26)

ART, antiretroviral therapy, HIV, human immunodeficiency virus, Hr-HPV, high risk human papillomavirus, IQR, interquartile range, Kg, kilogram, ml, milliliter, m^2^, square meter, WHO, World Health Organization, %, percentage.

### 3.3 Prevalence of Hr-HPV infection

### 3.4 Hr-HPV genotype profile among women living with HIV

Regarding genotype distribution, the majority of infections [76.21% (95% CI: 69.87%−81.56%)] were caused by other high-risk HPV types (non-16 and non-18). HPV-16 and HPV-18 infections were also frequently observed. Taken together, HPV-16 and/or HPV-18, whether alone or in co-infection, were detected approximately in 23,8% of Hr-HPV-positive participants. HPV-16 was detected in 6.8% of cases (95% CI: 4.05%−11.19%), while HPV-18 was present in 5.34% of cases (95% CI: 2.97%−9.43%). Coinfection of HPV-16 with other types was observed in 6.31% of cases (95% CI: 3.68%−10.61%), whereas coinfection of HPV-18 with other genotypes accounted for 4.85% (95% CI: 2.62%−8.83%). Finally, simultaneous coinfection of HPV-16, HPV-18, and other genotypes was present in only 0.49% of samples (95% CI: 0.06%−3.42%) (Figure 2).

**Figure 2:**
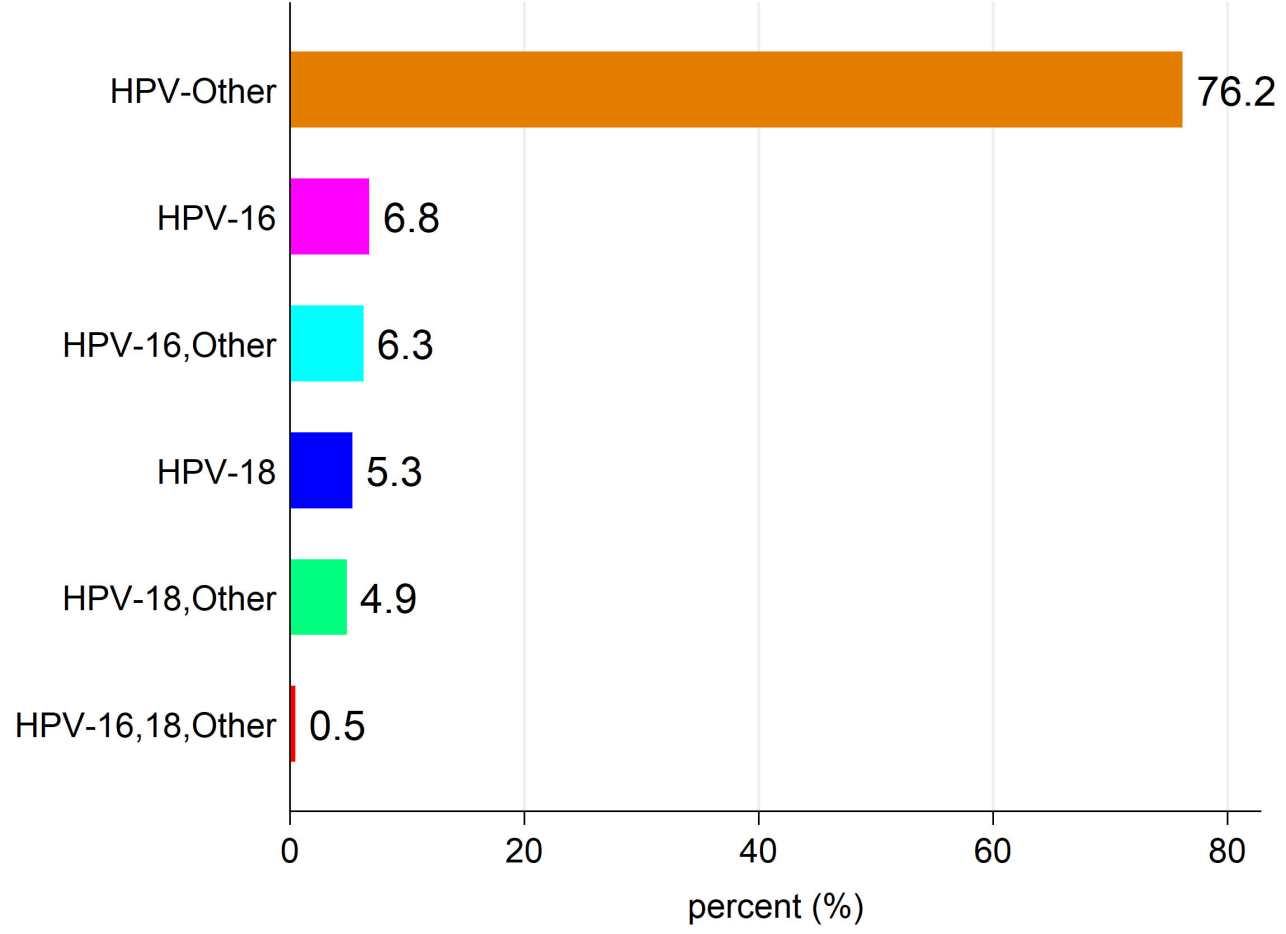
Hr-HPV genotype profile among women living with HIV, aged 25–65 years, under antiretroviral treatment at the DREAM Center in Kinshasa, DRC [*n* = 206].

### 3.5 Multivariable analysis

The prevalence of Hr-HPV infections in the study population was high, reaching 47.25% (206/436), with a 95% confidence interval (95% CI) of 42.58 %−51.96 % (Table 3).

**Table 3:** Prevalence of Hr-HPV among women living with HIV, aged 25–65 years, receiving antiretroviral therapy at the DREAM Center in Kinshasa, DRC [*n* = 436].

Hr-HPV	Frequency	% (CI 95%)
Negative	230	52.75 (48.04–57.42)
Positive	206	47.25 (42.58–51.96)

CI, confidence interval, Hr-HPV, high risk human papillomavirus, %, percentage.

#### 3.5.1 Assessment of multicollinearity

Assessment of multicollinearity showed VIF values ranging from 1.02 to 1.17, with a mean VIF of 1.08. All values were well below the predefined threshold of 5, indicating the absence of significant multicollinearity among the independent variables included in the model.

#### 3.5.2 Assesment of model fit

The Hosmer–Lemeshow goodness-of-fit test indicated good model fit (χ^2^(8) = 9.04, *p* = 0.339).

#### 3.5.3 Factors associated with Hr-HPV infection

In the univariate analysis, several variables such as marital status, HIV clinical stage, duration of antiretroviral therapy, and viral load, showed a significant association with Hr-HPV infection (Table 4).

**Table 4:** Associations between socio-demographic and clinical characteristics and HPV-Hr status in women living with HIV, aged 25 – 65 years, receiving antiretroviral treatment at the DREAM Center in Kinshasa, DRC [*n* = 436].

Variables	Status HPV (positive/total)	OR (CI95%)	*p*-value	aOR (CI95%)	*p*-value
Age range (years)					
25–29	15/25	1.00 (Ref.)	–	1.00 (Ref.)	–
30–39	55/111	0.65 (0.27–1.58)	0.347	0.86 (0.33–2.23)	0.758
40–49	74/169	0.52 (0.22–1.22)	0.133	0.64 (0.26–1.60)	0.342
50–59	50/109	0.56 (0.23–1.37)	0.206	0.79 (0.31–2.03)	0.628
60–65	12/22	0.80 (0.25–2.55)	0.706	1.15 (0.34–3.93)	0.820
Provenance					
Extrazonal	102/224	1.00 (Ref.)	–	–	–
Intrazonal (N'sele health zone)	104/212	1.15 (0.79–1.69)	0.462	–	–
Employment status					
None/unemployed	100/209	1.00 (Ref.)	–	–	–
Unskilled worker	85/179	0.99 (0.66–1.60)	0.943	–	–
Skilled worker	21/48	0.85 (0.45–1.59)	0.608	–	–
Level of education					
None or elementary	47/98	1.00 (Ref.)	–	–	–
High school	144/298	1.01 (0.64–1.60)	0.950	–	–
University	15/40	0.65 (0.31–1.38)	0.264	–	–
Marital status					
Single (single–divorced–widow)	135/259	1.63 (1.10–2.39)	**0.014**	1.43 (0.94–2.20)	0.098
In a relationship (married–cohabitation)	71/177	1.00 (Ref.)	–	1.00 (Ref.)	–
Age at first sexual intercourse (years)					
≤ 15	69/142	1.00 (Ref.)	–	1.00 (Ref.)	–
16–18	112/229	1.01 (0.67–1.39)	0.953	0.92 (0.59–1.43)	0.696
>18	25/65	0.66 (0.36 – 1.20)	0.175	0.62 (0.32–1.18)	0.146
Number of sexual partners (lifetime)					
<4	84/185	1.00 (Ref.)	–	1.00 (Ref.)	–
4–7	70/154	1.00 (0.65–1.54)	0.993	0.96 (0.60–1.53)	0.872
≥8	52/97	1.39 (0.84–2.27)	0.191	1.18 (0.69–2.02)	0.540
Contraceptive use					
Hormonal	25/52	1.04 (0.58–1.85)	0.898	1.19 (0.62–2.30)	0.594
None	181/384	1.00 (Ref.)	–	1.00 (Ref.)	–
Parity					
≤ 4	116/258	1.00 (Ref.)	–	1.00 (Ref.)	–
>4	90/178	1.25 (0.85–1.84)	0.250	1.34 (0.88–2.02)	0.171
BMI (Kg/m^2^)					
<18.5	31/49	1.89 (0.97–3.58)	0.051	1.54 (0.78–3.03)	0.210
18.5– <25	104/218	1.00 (Ref.)	–	1.00 (Ref.)	–
≥25– <30	48/112	0.82 (0.52–1.30)	0.403	0.78 (0.48–1.27)	0.316
≥30	23/57	0.74 (0.41–1.34)	0.322	0.73 (0.39–1.37)	0.329
WHO Clinical Stage (HIV)					
1 & 2	81/209	1.00 (Ref.)	–	1.00 (Ref.)	–
3 & 4	125/227	1.94 (1.32–2.84)	**0.001**	1.75 (1.16–2.64)	**0.008**
Duration of ART (Years)					
<2	40/65	1.98 (1.15–3.39)	**0.013**	1.91 (1.07–3.41)	**0.028**
≥2	166/371	1.00 (Ref.)	–	1.00 (Ref.)	–
HIV Viral load (copies/ml)					
<40	159/358	1.00 (Ref.)	–	1.00 (Ref.)	–
40– <1,000	27/50	1.47 (0.81–2.66)	0.204	1.50 (0.79–2.81)	0.212
≥1,000	20/28	3.13 (1.34–7.29)	**0.008**	3.13 (1.30–7.54)	**0.011**

ART, antiretroviral therapy, BMI, body mass index, CI, confidence interval, HIV, human immunodeficiency virus, HPV-Hr, high risk human papillomavirus, interquartile range, Kg, kilogram, ml, milliliter, m^2^, square meter, OR, odds ratio, aOR, adjusted odds ratio, *p*-value, probability value, WHO, World Health Organization, %, percentage. Bold values indicate statistically significant associations (*p* < 0.05).

In a multivariate analysis, only the association with HIV clinical stage, duration of antiretroviral therapy, and viral load remained statistically significative. The results suggest that women with advanced HIV disease (WHO clinical stage 3–4), a viral load ≥1,000 copies/ml, and duration of antiretroviral therapy of less than 2 years were more likely to be infected with Hr-HPV.

## 4 Discussion

This study was conducted to describe the characteristics of Hr-HPV in WLHIV in the city-province of Kinshasa, DRC. We estimated the prevalence and distribution of Hr-HPV infections and identified associated factors.

The results showed a particularly high prevalence of Hr-HPV infection in cervical samples collected using a cytobrush, with an overall frequency of 47.25% among women living with HIV in Kinshasa. This prevalence appears higher than those reported in studies conducted in the general population in Kinshasa, which range from 12.5 to 24.4% (11, 24, 25). However, these comparisons should be interpreted with caution, given differences in study populations, study settings, and methodologies. The higher prevalence observed in our study may partly reflect the specific characteristics of WLHIV, as HIV infection has been associated with an increased risk of Hr-HPV infection in several studies (6, 19, 21, 26, 27). Nevertheless, because our study did not include a concurrently recruited HIV-negative comparison group, we cannot directly quantify the difference in Hr-HPV prevalence associated with HIV status within the same study population. The higher burden of Hr-HPV infection among WLHIV is of particular concern given the increased risk of cervical cancer in this population. Globally, approximately 1 in 20 CCs is attributable to HIV; in Sub-Saharan Africa, approximately 1 in 5 CCs is due to HIV (7). The prevalence of Hr-HPV found in this study was, however, higher than that found in other studies in Africa among women living with HIV (28–33). It is, however, lower than that found in another study in Burkina Faso and South Africa (34). Such differences in Hr-HPV prevalence across African settings may reflect regional and population-level heterogeneity, as well as differences in study populations and methodologies (1, 12). Differences in cervical cancer incidence between countries may also be influenced by the availability and implementation of screening programs (1).

The results of this study also show that HPV-16 and HPV-18 types were detected in 23.8% of high-risk HPV positive participants, with nearly half of these infections corresponding to coinfections (11.65%), while the other half consists of isolated infections with HPV-16 or HPV-18 (12.14%). Furthermore, a substantial proportion of infections (76.21%) were associated with Hr-HPV other than types 16 and 18. These findings corroborate the results of other studies where a large proportion of infections were found to be related to Hr-HPV other than types 16 and 18. (28, 30–32) This observation is not surprising, as some studies in Africa have revealed distinct genotypic profiles, with types other than HPV-16 and HPV-18 predominating in certain populations at increased risk, such as women living with HIV (9, 34). Hence, the need for a more comprehensive approach to cervical cancer prevention and for studies providing a more detailed characterization of circulating HPV genotypes. The high prevalence of HPV-35 among reported women with confirmed cervical cancer in Zimbabwe further evidence of the diversity of HPV genotypes in sub-Saharan Africa (35). In our study, however, HPV-35 was included within the pooled “HPV-other” category and its individual frequency could not be determined. Similarly, the HPV genotype patterns reported among WLHIV in the region, characterized by a relatively lower prevalence of HPV-16 and a substantial contribution of other high-risk HPV types, highlight the importance of considering local epidemiological profiles when designing screening and prevention strategies (36). These findings also support the use of HPV testing approaches capable of detecting a broader range of high-risk HPV types and underscore the importance of tailoring screening and vaccination strategies to local epidemiological profiles. Such a comprehensive and context-specific approach is particularly relevant to achieving the WHO global strategy for the elimination of cervical cancer as a public health problem by 2030, which is based on the 90–70–90 targets: 90% of girls fully vaccinated against HPV by age 15 years, 70% of women screened using a high-performance test, and 90% of women identified with cervical disease receiving appropriate treatment and care. (6) The substantial proportion of non-16/18 Hr-HPV infections observed in our study also has implications for prevention. Although HPV-16/18 vaccination remains important, broader-valent vaccines, such as the 9-valent HPV vaccine, cover additional oncogenic types (31, 33, 45, 52, and 58) and may provide broader protection against HPV-related cervical disease. However, because the Abbott RealTime assay used in this study reports the non-16/18 Hr-HPV types as a pooled category, we could not determine the proportion of infections attributable to these individual vaccine-covered types. These findings support consideration of broader-valent HPV vaccination as part of comprehensive cervical cancer prevention strategies, particularly among women living with HIV, while recognizing that vaccination should complement rather than replace high-performance cervical cancer screening and appropriate treatment of precancerous lesions.

The results of this study further suggest that the HIV clinical stage, the duration of treatment antiretroviral therapy and HIV viral load were associated with Hr-HPV infection among WLHIV in Kinshasa.

Women with WHO clinical stages 3 and 4 had a significantly higher odds of Hr-HPV infection. Our results are similar to previous studies, which have reported an association between more advanced HIV disease and a higher prevalence of HPV infection (28, 37).

Also, women who have been on ARV treatment for less than 2 years was independently associated with Hr-HPV infection compared with a treatment duration of 2 years or more. These results corroborate those found in a study conducted in Burkina Faso and South Africa where people taking ARV for less than 2 years had a higher risk (34). Another study conducted in Ghana reported an association between highly active antiretroviral therapy and a lower risk of Hr-HPV infection (38). However, the association between antiretroviral treatment and cervical neoplasia remains poorly understood and is still debated, although ART in WLHIV is known to improve immune status (9). However, an alternative explanation should also be considered. Women with shorter ART duration may have been more recently diagnosed with HIV and may had pre-existing sexual or other behavioral characteristics associated with both HIV and HPV infection. However, although sexual and reproductive characteristics such as age at first intercourse and number of lifetime sexual partners were collected, information on sexual behaviors specifically preceding HIV diagnosis and on the timing of HIV diagnosis relative to HPV acquisition was not available. Therefore, this alternative explanation could not be directly assessed. In addition, WHO clinical stage and ART duration were considered as available indicators of HIV disease history and treatment exposure, but they do not fully capture the degree of immunosuppression.

Furthermore, in this study, among the 436 women with available VL data, 358 (82.1%) had an undetectable VL(<40 copies/ml), indicating good virological control. Women with a VL of at least 1,000 copies/ml had approximately threefold higher odds of Hr-HPV infection compared with women with an undetectable VL (adjusted OR = 3.13, 95% CI: 1.30–7.54; *p* = 0.011), although the relatively small number of women in this category (*n* = 28) warrants cautious interpretation. This finding is supported by several recent studies, including a systematic review and a meta-analysis, which highlight the importance of antiretroviral therapy (ART), CD4 count, and viral load in the risk of HPV infection and the development of cervical cancer (39). Furthermore, a study in pregnant women reported that those who achieved viral suppression were less likely to acquire Hr-HPV infection (40). These findings highlight the potential importance of adequate treatment adherence and viral suppression in relation to Hr-HPV infection (37, 39–41). Nevertheless, the absence of CD4 cell count data limited our ability to directly assess immunological status and means that residual confounding by immune status cannot be excluded.

### 4.1 Limitations

Although this study provides important information to inform health policies, it has certain limitations. First, the cross-sectional design, causal relationships between the identified factors and Hr-HPV infection cannot be established. Second, participants were recruited from a single specialized HIV care center, which may have introduced selection bias and may limit the generalizability of our findings to the broader population of WLHIV in Kinshasa. However, participants came from different areas of Kinshasa and were not exclusively from the area surrounding the study center. The center provides free and comprehensive HIV care, which may have reduced financial barriers to accessing care. Women receiving care at specialized centers may differ in socioeconomic characteristics, healthcare-seeking behaviors, access to care, and engagement in HIV care. Therefore, the findings should be interpreted in the context of the study population and generalized to the broader population of WLHIV in Kinshasa with caution. Third, monitoring of women receiving antiretroviral therapy was primarily based on HIV viral load measurements, while CD4 cell count data were not available. Although most women with available viral load data had an undetectable viral load, the absence of CD4 measurements prevented a direct assessment of immunological status. Therefore, the associations observed with WHO clinical stage and ART duration should be interpreted cautiously, as residual confounding by immune status cannot be excluded. However, information on smoking, history of sexually transmitted infections, adherence to antiretroviral therapy, and time since HIV diagnosis was not available, and these factors could not be included in the analysis. Their absence may have resulted in residual confounding and should be considered when interpreting the observed associations. In addition, the relatively small number of women with a viral load of at least 1,000 copies/ml may have limited the precision and stability of the corresponding association. Finally, the 12 high-risk HPV types other than HPV-16 and HPV-18 were reported as a single group by the Abbott RealTime assay, limiting a more detailed characterization of the genotype profile in this population. More comprehensive genotyping data would provide valuable information for guiding future HPV prevention and control strategies.

## 5 Conclusion

This study revealed a high prevalence of Hr-HPV infection, affecting approximately one in two WLHIV followed at the DREAM Center in Kinshasa. HPV-16 and HPV-18 were detected in arround one quarter of participants, while other high-risk HPV types accounted for the majority of infections. The analysis of associated factors showed that advanced HIV disease, unsuppressed HIV viral load, and a duration of antiretroviral therapy of less than 2 years were independently associated with Hr-HPV infection.

These findings highlight the need for regular cervical cancer screening and strengthened follow-up of WLHIV, including in populations benefiting from HPV vaccination. The substantial proportion of non-16/18 high-risk HPV types also underscores the potential value of broader HPV vaccine coverage, including higher-valent vaccines, as part of comprehensive cervical cancer prevention strategies.

## Data Availability

The raw data supporting the conclusions of this article will be made available by the authors, without undue reservation.
